# Comparative Analysis of Anthocyanin Compositions and Starch Physiochemical Properties of Purple-Fleshed Sweetpotato “Xuzishu8” in Desert Regions of China

**DOI:** 10.3389/fpls.2022.841969

**Published:** 2022-04-12

**Authors:** Hui Yan, Yungang Zhang, Muhammad Qadir Ahmad, Yaju Liu, Meng Kou, Meng Ma, Chen Li, Mohamed Hamed Arisha, Wei Tang, Xin Wang, Runfei Gao, Weihan Song, Zongyun Li, Qiang Li

**Affiliations:** ^1^Xuzhou Institute of Agricultural Sciences in Jiangsu Xuhuai District, Key Laboratory of Biology and Genetic Improvement of Sweetpotato, Ministry of Agriculture, Sweetpotato Research Institute, Chinese Academy of Agricultural Sciences, Xuzhou, China; ^2^Institute of Integrative Plant Biology, Jiangsu Key Laboratory of Phylogenomics and Comparative Genomics, School of Life Sciences, Jiangsu Normal University, Xuzhou, China; ^3^Department of Plant Breeding and Genetics, Bahauddin Zakariya University, Multan, Pakistan; ^4^Department of Horticulture, Faculty of Agriculture, Zagazig University, Zagazig, Egypt

**Keywords:** PFSP, anthocyanidins, anthocyanin compositions, arid environment, starch physicochemical properties

## Abstract

The present study was undertaken to determine the scope of sweetpotato cultivation in arid regions of China. For this purpose, we investigated yield, anthocyanin compositions and physicochemical properties of starch in purple-fleshed sweetpotato (PFSP) “Xuzishu8” under humid (zi8-X) and arid (zi8-D) environments of China. The experiment was conducted in three replications in both environments during 2019 and 2020. The yield and anthocyanidins contents of PFSP were significantly higher in the arid conditions as compared to humid. Zi8-X and zi8-D both revealed the presence of three anthocyanidins, namely, cyanidin (Cy), peonidin (Pn), and pelargonidin (Pg). Cy and Pn accounted for 36.40 and 63.54% of the total anthocyanidins in zi8-X, while in zi8-D, they were found as 26.13 and 73.80%, respectively. The quantitative analysis of these anthocyanins was performed using HPLC-ESI-MS/MS which revealed eighteen anthocyanins such as nine Cy, eight Pn and one Pg. Out of which, eleven anthocyanins showed a significant difference under both conditions. Starch and amylopectin contents were found to be increased by 15.39 and 4.71%, respectively, while the amylose concentration was reduced by 15.54% under the arid environment. The diameter of the starch granule and the peak viscosity were significantly higher under arid as compared to humid conditions. On the basis of results of this study, it seems quite practicable to develop PFSP cultivation in desert regions.

## Introduction

Purple-fleshed sweetpotato (PFSP) is a widely cultivated crop in China owing to its rich nutritional value, high yield, and its ability to adapt to adverse environmental conditions. Sweetpotato, ranks at 4th position in crop cultivation after rice, wheat, and corn (Pablo et al., 2019). China, being the biggest sweetpotato producer, accounts for 63.87% of the total crop production and contributes to 36.65% of the world agronomy (Food and Agriculture Organization, 2017). Concurrently, China is endowed with a wide range of arid areas, especially the desert in Dalad Banner, Inner Mongolia (Li and Niu, 2019), which can be utilized for sweetpotato cultivation.

The storage roots of PFSP are the storehouse of anthocyanidins and starch. Anthocyanidins not only act as excellent natural food additives and pigments, but also have a health-promoting role in cardiovascular diseases, hyperlipidemia, and have pronounced antioxidant properties (Oki et al., 2003; Steed and Truong, 2010; Li et al., 2017; Tamara et al., 2019). With the enhanced usage of PFSP as raw materials in anthocyanin extraction, starch processing, healthcare products, and cosmetics industry, the demand of PFSP is increasing rapidly (Hock et al., 2017).

Anthocyanin and starch are two important traits effecting quality and utility of PFSP. Previously, researchers had reported that the stability and functionality of anthocyanins vary dramatically which effects the processing quality and utility (Bolivar and Luis, 2004; Truong et al., 2010; Gras et al., 2017). Previous studies revealed that anthocyanin synthesis was temperature-dependent and was inversely proportional to the rise in temperature (Mori et al., 2005; Zeeman et al., 2010; Zhou et al., 2015; Lila et al., 2018). However, there were very few studies that investigated the variations of anthocyanin contents and compositions of PFSP under arid environment. The “Xuzishu 8” is a newly developed PFSP variety with high anthocyanin content and high yield (Arisha et al., 2021; Yan et al., 2022), but its anthocyanin compositions and contents under different environmental milieu are still unknown. Therefore, in order to test the feasibility of planting PFSP in arid land, it is of considerable significance to explore the anthocyanin compositions of PFSP in desert area.

The concentration of the starch and its physiochemical properties are important indexing parameters to evaluate the attributes of starch (Tian et al., 1991; Sun et al., 2018; Wang et al., 2020; Ding et al., 2021). The concentration of starch constituents can vary considerably by 10% under different ecological environments (Tian et al., 1991). Most of the earlier studies have mainly been focused on the variations in starch under different environments and overlooked to analyze the physicochemical properties of starch, such as starch granule diameter (DG), chain length distribution of amylopectin (DP), and starch pasting properties [Rapid Visco Analyser (RVA)], which are important parameters for evaluating processing characteristics. These properties of starch determine its distinctive applications in food processing and bio-industry. Therefore, a more in-depth research is needed for understanding the attributes of starch, which will guide better yield.

## Materials and Methods

### Field Trials and Storage Root Harvesting

The experiment was carried out at two different regions such as Xuzhou (Jiangsu eastern China) and Dalad Banner (northwest of Ordos plateau). Xuzhou has temperate monsoon climate which is suitable for sweetpotato cultivation, whereas Dalad Banner has typical temperate continental climate where available water for agriculture is limited. Dalad Banner located in Ordos plateau, is a typical desert land representing soil and ecological status of Inner Mongolia (Zheng, 2020). The annual average precipitation in Dalad Banner was about 300 mm, and 75% of which occurred during June to September (Zheng, 2020). While in Xuzhou, the annual average precipitation was 800–930 mm.

The storage roots of PFSP *Ipomoea batatas* (L.) Lam. cv. “Xuzishu8,” were obtained from the Xuzhou Institute of Agricultural Sciences, Jiangsu, Xuhuai District, China. These storage roots were sown in the soil at room temperature approximately 30°C and relative humidity 90% for germination. After 20 days of sowing, germinated seedlings were cut from the storage roots and transferred on the raised beds. After 25 days, these plants were transplanted in the field separately at Xuzhou (Latitude: 34° 12′ 1″ N, longitude: 117° 9′ 54″ E, altitude: 135 m) and Dalad Banner (Latitude: 40° 12′ 25.80″ N, Longitude: 109° 57′ 24.59″ E, altitude: 1,009 m) experimental stations (soil composition and weather information were shown in Supplementary Tables 1, 2). The experiment was conducted in Randomized Complete Block Design (RCBD) and replicated thrice for two consecutive years 2019 and 2020. The plants were planted on seven rows 5.6 m × 6.0 m plot in each replication. Row to row and plant to plant distance were maintained as 80 and 25 cm, respectively.

The crop at Dalad Banner and Xuzhou, received one irrigation at the time of transplantation only. Sweetpotato plants showed vigorous growth approximately 60 days after transplantation and started formation of root tubers at both locations. For yield determination, after 136 days to planting, storage roots from 100 sweetpotato plants from inner five rows measuring 4.0 m × 5.0 m (20 m^2^) were sampled leaving border rows to exclude border effect. The storage roots from these selected plants were harvested, washed thoroughly, and weighed to determine plot yield (kg/20 m^2^).

### Determination of Yield and Dry Matter Contents

The yield (kg/ha) was determined by the formula given below and then the data from each replication was averaged (Yan et al., 2019).

yield (kg/ha) = Plot yield (kg/20 m^2^) × 500.

The dry matter contents (DM) were determined by following oven-drying method. For this purpose, 100 g fresh storage root samples were taken from five randomly harvested plants, separately. These samples were cut into small pieces and then oven dried for 10 h at 75°C until reached at a constant weight (M/g). After this the dried samples were grinded into homogenous fine powder. Dry matter content was calculated by following formula and the data were averaged for each replication.

DM (%) = M/100 g × 100%.

### Determination of Total Anthocyanidin Content and Anthocyanidin Yield

For the determination of anthocyanin contents, storage roots from 5 randomly selected plants were cut into small pieces, freeze-dried, and stored separately. These storage roots were used for the determination of total anthocyanin contents, yield, and quantification.

Total anthocyanidin content was measured by the modified method of Tang et al. (2018). For this purpose, 1 g freeze-dried samples of storage roots was taken from each plant separately and extracted in citric acid-disodium hydrogen phosphate buffer (pH = 3.0) in 50 ml centrifuge tube. Samples were crushed in a capacity wearing commercial blender for 30 s until the residues turned white, then the solution was diluted to 50 ml and kept for 30 min. The extracts were centrifuged at 4,000 rpm for 8 min. The absorbance of the supernatant was determined at a wavelength of 530 nm using a spectrophotometer (UV 3200, Shanghai, China).

The total anthocyanidin content was calculated using the following formula (Tang et al., 2018):

The total anthocyanidin content (mg/100 g fresh weight) = 1/958 × 1 g × V (ml)/100 × Abs_530_ × 100,000

The yield of total anthocyanidins was calculated with the following formula:

Yield of total anthocyanidins (kg/ha) = Yield of PFSP (kg/ha) × The total anthocyanidin content (mg/100 g fresh weight)/100,000

### Extraction, Separation, and Quantification of Anthocyanins

The anthocyanins extracted from the freeze-dried storage roots were analyzed using a Liquid Chromatography-Electrospray Ionization-Tandem Mass Spectrometry (LC-ESI-MS/MS) system (HPLC, UFLC SHIMADZU CBM30A system, MS, Applied Biosystems 4500 QTrap) (Wang et al., 2018). Twenty one standards developed by ChromaDex company name cyanidin 3-(6″-caffeoylsophoroside)-5-glucoside, cyanidin 3-p-hydroxybenzoylsophoroside-5-glucoside were used for quantification of anthocyanins (Supplementary Table 3).

#### Anthocyanin Extraction

For anthocyanin extraction, 1 g freeze-dried storage roots sample was taken from each plant separately, and extracted with 10 ml extraction solution (extraction solution was prepared by mixing 50 ml acetone, 50 ml methanol and 10 ml formic acid). Anthocyanins were determined by ultrasonic method and the solution was kept at room temperature for 1 h and shaken 2–3 times followed by centrifugation at 6,000 rpm for 20 min at 4°C in a refrigerated centrifuge. The supernatant was transferred into a 100 ml rotating bottle and condensate using a rotary evaporator (Model, Tokyo physicochemical company, Japan, N-1300D-WB) until the extract mixture was concentrated to 2 ml. The temperature of the water bath pot was kept at 40°C. Then 800 μl extract was transferred to sample loading bottle prior to HPLC-ESI-MS/MS analysis (Wang et al., 2018).

#### LC-MS for Separation and Quantitation of Anthocyanins

For each plots, three biological replications were analyzed, independently. The sample extracts were analyzed using a Liquid Chromatography-Electrospray Ionization-Tandem Mass Spectrometry (LC-ESI-MS/MS) system (HPLC, UFLC SHIMADZU CBM30A system). Briefly, the HPLC system consisted of Waters ACQUITY UPLC HSS T3 C18 column (1.8 μm, 2.1 mm,⋅100 mm). Elution was performed with the solvent A Methanol/acetonitrile (1:1), and deionized water as solvent B. The gradient program was as follows: 0–10 min, 50% B; 10–15 min, 80% B; 15–24 min, 100% B; 24–30 min, 100% B; 31 min, 50% B. Flow rate, temperature and injection volume was 0.40 ml/min, 40°C and 5 μl, respectively. The effluent was alternatively connected to an ESI-triple quadrupole-linear ion trap (Q TRAP)-MS.

Linear ion trap (LIT) and triple quadrupole (QQQ) scans were acquired on a triple quadrupole-linear ion trap mass spectrometer (QTrap), API 4500 QTrap LC/MS/MS system. TSI ion source was in the positive ion mode. The ion source voltage and the source temperature were as 5,500 V and 600°C, respectively.

The mass spectra in the range m/z 50–1,500 were obtained by electrospray ionization in positive-ion mode (Wang et al., 2018).

### Determination of Starch and Sugar Contents

#### Separation and Purification of Starch

Starch was extracted according to the method of Zhao et al. (2011) and Amanda et al. (2018). For this purpose, fifteen plants were randomly selected. For sample preparation, storage roots from five plants (out of fifteen) were pooled separately, and a representative sample of 10 kg was weighed. Overall, three samples of 10 kg storage roots each were prepared.

The storage roots were thoroughly washed in tap water and crushed into a commercial blender. The slurry was filtered through a 100 μm sieve. The starch was allowed to settle for 5–6?h and the supernatant was decanted off, then the starch was dried in an oven with a fan-forced ventilation at 40°C for 2 days. The starch content was determined using the following formula:

Total starch contents [%(DW)] = Weight of starch/(10 kg × DM) × 100%

#### Determination of Amylose Content

The amylose content of PFSP was determined by following Xia et al. (2006). Briefly, 50 g starch from each sample as described earlier, was added into the dispersed solution (400 ml dimethyl sulfoxide [DMSO] and 100 ml deionized water), separately. The solution was kept at 25°C for 4.5 h and stirred until an emulsion was formed. Then the emulsion was placed in a water bath at 60°C for 30 min then 100 ml of *N*-butanol was added to the solution followed by continuous stirring for 5 min. After this, solution was allowed to cool to room temperature. Then the solution was centrifuged at 2,000 rpm for 30 min. The supernatant was taken into a 500 ml flask and 200 ml of 1% NaCl solution was added to the flask followed by constant heating at 80°C for 30 min until the precipitate was completely dissolved. Then 100 ml of *N*-butanol was added to the hot solution slowly and stirred with a glass rod then the flask was withdrawn from the water bath and cooled to the room temperature. Now the solution was centrifuged at 2,000 rpm for 30 min and the supernatant was transferred to a petri dish and dried at 70°C in a vacuum oven until amylose was obtained by constant weight.

The concentration of amylose was determined by using the colorimetric method (Knutson and Grove, 1994; Xia et al., 2006). For this purpose, a standard work curve was made where y = 113.13x - 8.5821, R^2^ = 0.9971.

For the determination of OD value, 50 mg of amylose from each sample was added to 2 ml of 90% DMSO and dissolved at 80°C. Firstly, diluted this solution to 100 ml, then take 2 ml of starch solution and measure the OD value at 720 nm. Then the amylose content was calculated according to the OD value and standard curve.

#### Concentration of Soluble Sugars

The concentration of soluble sugars such as glucose, sucrose, and fructose were determined by HPLC-ELSD (evaporative light scattering detector) (Shen et al., 2020). For this purpose, 1.0 g storage root samples were taken from five randomly harvested plants, separately. Each sample was homogenized with 5 ml of water using mortar and pestel. The homogenized material was transferred to an eppendorf tube and kept at room temperature for 2 h followed by vertexing. Then the solution was centrifuged at 4,000 rpm for 10 min. Now, 1 ml of supernatant was taken in a separate tube and 3 ml of acetonitrile was added to the solution followed by filtering with 0.22 μm microporous membrane for chromatographic analysis.

The chromatographic analysis for the determination of soluble sugar content was performed using HPLC-ELSD (AllChrome ELSD 6000). For this purpose, briefly, PrevailtmCarb ES coumn-W 250 mm × 4.6 mm was used for stationary phase and for mobile phase acetonitrile-water (volume ratio 65:35) was used with the flow rate 0.8 ml/min. Column temperature: 30°C, drift tube temperature: 95°C, nitrogen flow: 2.4 ml/min, and injection volume: 4.0 μl.

Smooth unimodal distribution of DG was demonstrated by zi8-X and zi8-D variety (Figure 3A). However, there was an evident dissimilarity present in the DG distribution and average DG. The data generated from laser scattering particle analyzer revealed that DG of zi8-X ranged from 8.30 to 27.17 μm, while in zi8-D it was from 9.84 to 31.13 μm. Additionally, the average DG of zi8-X was 16.88 μm which was significantly different when compared to zi8-D whose average DG was 19.56 μm (*p* < 0.05).

**FIGURE 1:**
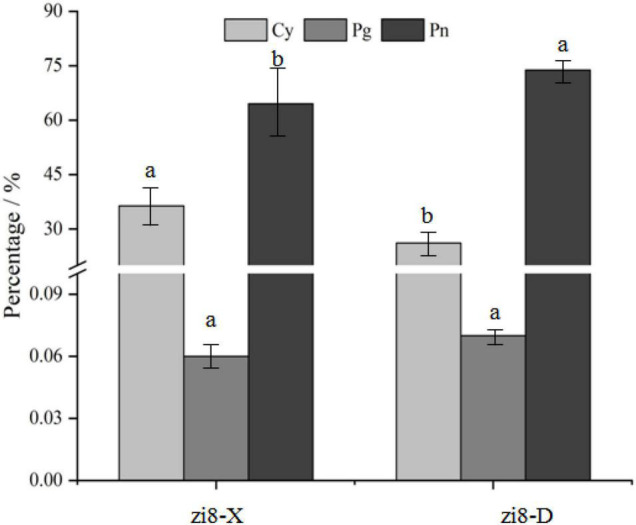
Quantitative of anthocyanidins and their percentage in zi8-X and zi8-D.

**FIGURE 2:**
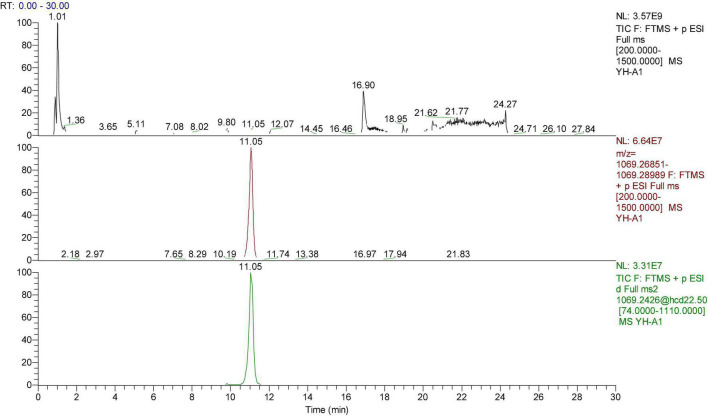
The HPLC-ESI-MS/MS of peonidin 3-caffeoyl-p-hydroxybenzoyl-sophoroside-5-glucoside. Upper chromatograms: Total ion chromatogram (TIC); Middle chromatograms: Extracted Ion Chromatogram (EIC); Bottom chromatograms: Secondary fragment diagram.

**FIGURE 3:**
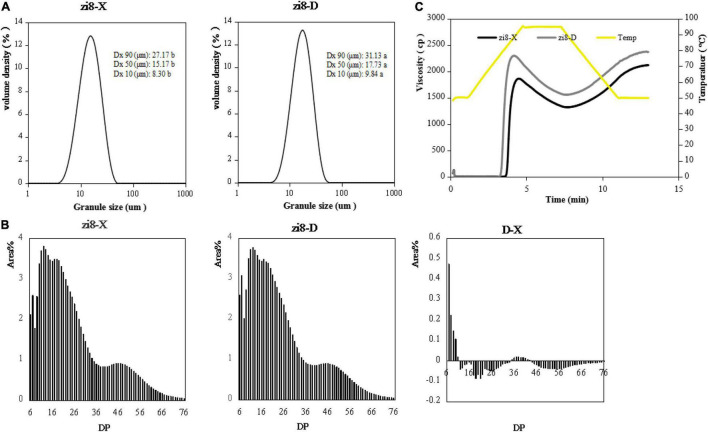
Physicochemical properties of starch from zi8-X and zi8-D. **(A)** Particle size distribution; **(B)** Curves of chain length distributions of zi8-X and zi8-D; **(C)** RVA pasting profiles of zi8-X and zi8-D.

#### Assessment of Starch Physiochemical Properties

Following starch, physiochemical properties were determined from starch and amylase contents determined in the previous experiment. The amylose was separated as described earlier. The particle size distribution was determined using a Mastersizer 3000 laser diffraction instrument (Malvern Instruments Ltd., Worcestershire, United Kingdom) in wet-cell mode (Zhou et al., 2015). A laser scattering particle analyzer (Malvem Mastersizer 2000, United Kingdom) was employed to ascertain the starch DG. DG was further classified based on the grading method of Shi et al. (2011). For this purpose, 50 mg samples were dispersed with absolute ethanol and ultrasonic for 30 s. The DG was represented by D10, D50, and D90 (D10, D50, and D90, respectively, represent the particle size with cumulative particle distribution of 10%, median particle size, and cumulative particle distribution of 90%).

Amylopectin chain length distribution is called the degree of polymerization (DP). DP of amylopectin was analyzed utilizing high-performance anion-exchange chromatography with a pulsed amperometric detector (HPAEC-PAD; Dionex-ICS 3000; Dionex Corporation, Sunnyvale, CA, United States) (Zhou et al., 2015). For this purpose, 50 mg starch sample from each experiment was extracted with 5 ml of acetic acid sodium acetate (0.05 mol/L, pH = 3.5) buffer solution. Then the sample was kept in boiling water for 20 min in a water bath to completely gelatinize, then the solution was cooled at room temperature. After this, 20 μl isoamylase was added into the solution and kept in a water bath at 37°C constant temperature for 48 h. After this, the solution was boiled 20 min in water bath to inactivate the enzyme. Then the solution was filtered with 0.45 μM filter membrane followed by 10 times dilution. Finally, the solution was filtered with 0.22 μM filter membrane. Now the samples were analyzed by ion chromatography.

Pasting property of the PFSP starches was measured using a Rapid Visco Analyser (Model RVA-4C; Newport Scientific Pty. Ltd., Warriewood, NSW, Australia). Starch sample (3 g) extracted from each tagged plant was dissolved into 25 ml distilled water. Temperature profile for RVA was kept as: initially samples were kept at 35°C for 5 min followed by heating at 93°C for 10 min. Then the sample was cooled down at 35°C for 3 min (Zhou et al., 2015).

A method using Gel Permeation Chromatography (GPC) equipped with multi-angle laser-light scattering (MALLS) and refractive index detector (RID, Waters HMW 6E) was presented for determining molecular features of starch, such as molar mass, polydispersity, RMS, and configuration plot. The parameters are as follows: Mobile phase: NaNO_3_; Flow rate: 0.4 ml/min; Column temperature: 60°C; Analytical column model: waters HMW 6E; Sample loading: 100 μl.

### Statistical Analysis

Data were expressed as mean ± *SD*. The Duncan test and one-way analysis of variance (ANOVA) were used for multiple comparisons by SPSS 13.0 software package (IBM, United States). Difference was considered to be statistically significant if *p* < 0.05.

## Results

### The Yield and Total Anthocyanidin Content

The yield and total anthocyanidins content of PFSP were significantly higher in the arid area as compared to humid area. The yield and dry matter content of zi8-D were 47,060 kg/ha and 32.09% as compared to 38,362.5 kg/ha and 28.09% of zi8-X which was 22.67 and 14.24% higher, respectively. Total anthocyanidin content of zi8-D was remained 125.19 mg/100 g FW as compared to 89.48 mg of zi8-X, which was 39.91% higher. The yield of total anthocyanidins of zi8-D was 58.75 kg/ha, which was 73.66% higher (Table 1).

**TABLE 1:** The total anthocyanidins content and yield of anthocyanidins.

Year	2019		2020		Average	
	
Trait	zi8-X	zi8-D	zi8-X	zi8-D	zi8-X	zi8-D
Yield of PFSP (kg/ha)	39,300 ± 418.32	48,500 ± 857.54*	37,425 ± 524.30	45,620 ± 749.50*	38362.5	47060
Dry matter content (%)	27.53 ± 1.22	32.28 ± 0.97*	28.65 ± 0.75	31.89 ± 1.06*	28.09	32.09
The total anthocyanidins content (mg/100 g FW)	84.21 ± 4.28	113.52 ± 3.92*	94.75 ± 3.24	136.85 ± 4.96*	89.48	125.19
Yield of total anthocyanidins (kg/ha)	33.09 ± 2.98	55.06 ± 3.64*	35.46 ± 3.03	62.43 ± 3.35*	33.83	58.75

*Significant at p < 0.05.

*FW, fresh weight.*

### Quantitative Determination of Anthocyanidins

As shown in Figure 1, zi8-X and zi8-D both revealed the presence of three anthocyanidins, namely, cyanidin (Cy), peonidin (Pn), and pelargonidin (Pg). Cy and Pn accounted for 36.40 and 63.54% of the total anthocyanidins in zi8-X, while in zi8-D, they were found as 26.13 and 73.80%, respectively. The Pg content was found to be 0.06%, which was extremely low. Overall, it was observed that Cy contents were 39.30% lower, and Pn contents were 16.15% higher in zi8-D than zi8-X (*p* < 0.05) (Figure 1).

### Quantitative Determination and Variation Analysis of Cyanidin, Peonidin, and Pelargonidin

Eighteen anthocyanins were identified by HPLC-ESI-MS/MS in zi8-X and zi8-D, which includes nine cyanidins, eight peonidins, and one pelargonidin (Table 2). The same anthocyanins were revealed in both the samples, but eleven of them showed a significant difference in percentage when tested under the arid and humid environments (*p* < 0.05). The highest content of cyanidin was depicted by cyanidin 3-(6″-caffeoylsophoroside)-5-glucoside (No. 1), which accounted for 15.49 and 6.07% in zi8-X and zi8-D, respectively, followed by cyanidin 3-p-hydroxy benzoyl sophoroside-5-glucoside (No. 2). Interestingly, except cyanidin 3-p-hydroxy benzoyl sophoroside-5-glucoside, all the other eight cyanidins showed higher value in zi8-X, out of which, five were significant at *p* < 0.05 (No. 1, No. 3, No. 5, No. 8, and No. 9). The percentage of cyanidin 3-p-hydroxy benzoyl sophoroside-5-glucoside (No. 2) was found to be 58.65% higher in zi8-D than zi8-X (*p* < 0.05). These results were consistent with Figure 1.

**TABLE 2:** The identification and quantification of Cy, Pn, and Pg from zi8-X and zi8-D.

	No.	zi8-X (%)	zi8-D (%)	No.	zi8-X (%)	zi8-D (%)
**Cy**	1	15.49 ± 0.56*	9.21 ± 0.65	2	6.07 ± 0.42	9.63 ± 0.41*
	3	6.02 ± 0.63*	1.82 ± 0.15	4	4.66 ± 0.37	3.26 ± 0.34
	5	2.13 ± 0.32*	0.77 ± 0.14	6	1.75 ± 0.12	1.27 ± 0.21
	7	0.21 ± 0.04	0.18 ± 0.02	8	0.06 ± 0.01*	0.01 ± 0.00
	9	0.02 ± 0.01*	0.01 ± 0.00	**Cy**	**36.40**	**26.13**
**Pn**	10	23.95 ± 0.83	35.50 ± 1.65*	11	17.30 ± 0.76*	12.68 ± 0.49
	12	7.84 ± 0.23	16.14 ± 0.75*	13	5.57 ± 0.23	4.97 ± 0.23
	14	5.27 ± 0.53*	1.95 ± 0.16	15	1.71 ± 0.01	1.29 ± 0.12
	16	1.26 ± 0.07*	0.63 ± 0.01	17	0.66 ± 0.13	0.64 ± 0.12
	/	/	/	**Pn**	**64.53**	**73.80**
**Pg**	**18**	**0.06** ± **0.00**	**0.06** ± **0.00**			

*Significant at p < 0.05. No.1Cyanidin 3-(6″-caffeoylsophoroside)-5-glucoside; No.2Cyanidin 3-p-hydroxybenzoylsophoroside-5-glucoside; No.3 Cyanidin3-(6″-caffeoyl-6″′-feruloylsophoroside)-5-glucoside; No.4 Cyanidin 3-sophoroside-5-glucoside; No.5Cyanidin 3-(6″,6″′-dicaffeoylsophoroside)-5-glucoside; No.6 Cyanidin 3-(6″-feruloylsophoroside)-5-glucoside; No.7 Cyanidin 3-(6″-p-coumarylsophoroside)-5-glucoside; No.8 Cyanidin 3-caffeoylsophoroside-5-glucoside; No.9 Cyanidin 3-caffeoyl-p-coumarylsophoroside-5-glucoside; No.10 Peonidin 3-caffeoyl-p-hydroxybenzoyl-sophoroside-5-glucoside; No.11 Peonidin 3-caffeoylsophoroside-5-glucoside; No.12 Peonidin 3-p-hydroxybenzoylsophoroside-5-glucoside; No.13 Peonidin 3-sophoroside-5-glucoside; No.14 Peonidin 3-feruloyl-p-caffeoylsophoroside-5-glucoside; No.15 Peonidin 3-(6″-feruloylsophoroside)-5-glucoside; No.16 Peonidin 3-(6″,6″′-dicaffeoylsophoroside)-5-glucoside; No.17 Peonidin 3-caffeoylsophoroside-5-glucoside; No.18 Pelargonidin 3-sophoroside-5-glucoside. Cy, Cyanidin; Pn, Peonidin; Pg, Pelargonidin.

The peonidin 3-caffeoyl-p-hydroxybenzoyl-sophoroside-5-glucoside (No. 10, Figure 2) was proved to be the most abundant peonidin in “Xuzishu8,” which represented 23.95 and 35.50% of the total anthocyanins in zi8-X and zi8-D, respectively. Two peonidins (Nos. 10 and 12) were found to be significantly higher in zi8-D, while peonidin numbers 11, 14, and 16 were significantly higher in zi8-X. The results revealed that although the composition of anthocyanin was dependent on inheritance traits, their contents are widely affected by environmental factors.

### Evaluation of Starch and Sugar Contents

The significant differences were observed in the starch and soluble sugar contents between zi8-X and zi8-D (*p* < 0.05). Under the arid environment, the percentage of total starch and amylopectin were higher by 15.39 and 4.71% in zi8-D, respectively, as compared to zi8-X. Whereas, zi8-D exhibited reduction in the amylose contents by 13.45%. Three soluble sugars contents were higher in zi8-X, and sucrose and fructose arrived significance level (*p* < 0.05) (Table 3).

**TABLE 3:** Variations in the starch and soluble sugars contents of zi8-X and zi8-D (mean ± SE).

Traits	zi8-X	zi8-D
Total starch contents [%(DW)]	55.36 ± 0.78	63.88 ± 0.67*
Amylose contents [%(DW)]	25.95 ± 0.66*	22.46 ± 0.59
Amylopectin contents [%(DW)]	74.05 ± 0.06	77.54 ± 2.59*
Amylose/Amylopectin ratio	0.35 ± 0.02*	0.29 ± 0.01
Glucose (mg/g, FW)	37.62 ± 2.50	28.78 ± 3.04
Sucrose (mg/g, FW)	37.8 ± 3.10*	29.51 ± 0.98
Fructose (mg/g, FW)	175.48 ± 0.96*	147.25 ± 7.26

*Significant at p < 0.05.

### Variation Analysis of Starch Physiochemical Properties

The physicochemical properties of starch consist of starch DG, the chain length distribution of amylopectin (DP), RVA profiles and molecular features. All of these properties exhibited quite different outcomes when compared between zi8-X and zi8-D (Figure 3).

The DP of zi8-X and zi8-D exhibited analogous curves (Figure 3B). According to the degree of polymerization, the chain length distribution of amylopectin is divided into four parts: DP 6-15, DP 16-25, DP 26-35, DP > 36. Both zi8-X and zi8-D revealed the chain length at 6 and displayed three peaks at around 12, 17, and 45. Though there was no significant difference between zi8-X and zi8-D in average DP, zi8-D exhibited a higher number of short chains and a lower number of long chains under the arid environment (Table 4).

**TABLE 4:** Chain length distributions in zi8-X and zi8-D (mean ± SE).

DP	zi8-X (%)	zi8-D (%)
DP 6-15	30.78 ± 2.13	32.12 ± 2.41*
DP 16-25	31.45 ± 1.75	30.94 ± 3.55
DP 26-35	16.05 ± 1.37	15.82 ± 1.33
DP > 36	21.71 ± 2.61	21.12 ± 1.94
Average DP	35.42 ± 3.02	34.80 ± 2.82

*Significant at p < 0.05.

The starch pasting profiles of zi8-X and zi8-D exhibited similar results (Figure 3C). However, the parameters of viscosity, including peak viscosity, final viscosity, peak time and pasting temperature, were found to be significantly different (*p* < 0.05) as represented in Table 5. The pasting temperature of zi8-X was 6.66% higher than zi8-D (76.60°C), while the zi8-D showed significantly higher peak viscosity and final viscosity as 22.01 and 11.67% (*p* < 0.05), respectively.

**TABLE 5:** Viscosity properties of “Xuzishu8” starch from the different environments.

Sample*^a^*	Peak viscosity (cP)	Trough1 (cP)	Breakdown (cP)	Final visc (cP)	Setback (cP)	Peak time (min)	Pasting temp (°C)
zi8-X	1,875 ± 3	1,339 ± 2	536 ± 5	2120.30 ± 8.70	781.3 ± 10.6	4.47 ± 0	81.70 ± 0.05*
zi8-D	2287.70 ± 14.30*	1549.70 ± 11.30*	738 ± 3.20*	2367.70 ± 10.70*	818.00 ± 3.6*	4.13 ± 0*	76.60 ± 0.02

*Significant at p < 0.05; ^a^Values represent “mean ± Standard error”.

**FIGURE 4:**
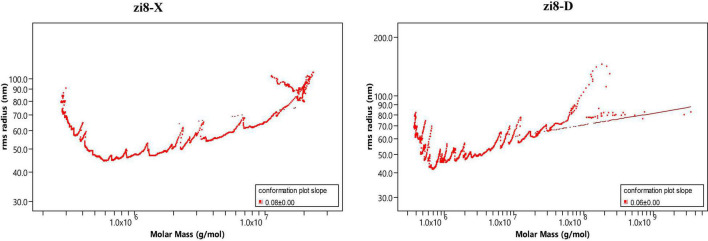
R.M.S conformation plot.

The weight-average mother weight (Mw), number-average molar mass (Mn), Z-average molar mass (Rz), and Polydispersity (PDI) were obtained using the GPC-RI-MALS system (Table 6). Molecular features of starch showed a great difference between zi8-X and zi8-D. Mw (Rz) of zi8-X and zi8-D starch were measured to be 23.2 × 10^5^g/mol (72.4 nm) and 35.1 × 10^5^ g/mol (71.2 nm), respectively. PDI was usually represented by ratio Mw/Mn, PDI of zi8-D was higher than zi8-X. The polymer configuration of “Xuzishu8” was determined according to the conformation plot (Figure 4). Taking log (molar mass) as abscissa and log (R.M.S. radius) as ordinate, the slope indicated that the molecular configuration of polymer was different between zi8-X and zi8-D.

**TABLE 6:** Determination of molecular features of sweetpotato “Xuzishu8” by GPC-RI-MALLS system.

Molecular features		Peak (zi8-X)	Peak (zi8-D)
Molar mass (× 10^5^g/mol)	Mw	23.2 (± 1.6%)	35.1 (± 2.2%)*^a^*
	Mn	6.4 (± 1.2%)	8.3 (± 1.4%)
	Mz	103.3 (± 4.7%)	661.2 (± 7.1%)
Polydispersity (PDI)	Mw/Mn	3.6 (± 2.0%)	4.2 (± 2.6%)
	Mz/Mn	16.2 (± 4.8%)	79.5 (± 7.3%)
R.M.S (nm)	Rw	57.6 (± 2.9%)	54.2 (± 3.7%)
	Rn	59.0 (± 2.9%)	55.6 (± 3.8%)
	Rz	72.4 (± 2.8%)	71.3 (± 4.0%)

*^a^Values represent “mean ± Standard error”.*

## Discussion

In the present study, we successfully planted PFSP in desert areas of China and investigated the yield, total anthocyanidins, anthocyanin compositions, and physicochemical properties of starch of “Xuzishu8” in desert regions. This study proved that it is quite practicable to develop PFSP cultivation in desert regions.

The two trials held at Xuzhou and Dalad Banner had experienced great variation of precipitation and temperature. The growing season of PFSP is usually from June to October in northern China. During this period, Xuzhou experiences rainfall with increased temperature and humidity in the air, whereas Dalad Banner faces relatively cool and dry weather. So, the variation in temperature and rainfall was probably the utmost possible factors which caused the qualitative variation in PFSP.

The effect of temperature on anthocyanin accumulation mainly plays a role in two aspects. One is that appropriate low temperature promotes the expression of regulatory genes and structural genes, so as to promote the accumulation of anthocyanin, for example, *Myb* expression increased induced by low temperature (Ban et al., 2007). On the other side, high temperature affected the stability of anthocyanin then reduced its accumulation (Ban et al., 2007). The studies have done by Mori et al. (2005); Huh et al. (2008) and Gao et al. (2016) revealed that high temperature inhibits the accumulation of anthocyanin.

One of the major disadvantages of the aforementioned studies was that the effect of precipitation was studied less extensively (Ban et al., 2007; Jeny et al., 2020). In the present investigation, total anthocyanidin concentration was increased by 39.94% in zi8-D when compared to zi8-X. Pn and Cy were observed as the major anthocyanidins of “Xuzishu8,” and accounted for 73.80 and 26.13% in zi8-D, respectively. In zi8-D, Pn content was increased by 14.37% when compared to zi8-X. Based on the results, it can be stated that drought and low temperature are the factors that might contribute to the accumulation of total anthocyanidins and Pn. However, extensive research is required to know the role of precipitation and temperature, affecting the concentration of anthocyanidins and Pn.

Different cultivars of PFSP have varied compositions of anthocyanins (Terahara et al., 2004; Truong et al., 2010; Kim et al., 2012; Xu et al., 2015; He et al., 2016). Previously, seventeen anthocyanins have been identified in “Stokes Purple” and “NC 415,” whereas in “Guangzi1,” only nine anthocyanins have been observed (He et al., 2016),and twelve anthocyanins identified in “P40” (Xu et al., 2015). In the present study, for the first time, eighteen anthocyanins were identified in “Xuzishu8” out of which eleven showed a significant difference between arid and humid regions. The results revealed that although the composition anthocyanin was hereditary, their contents are highly dependable on the environmental interactions.

It has been reported that the stability and functionality of anthocyanins vary dramatically (Bolivar and Luis, 2004; He et al., 2016). Cyanidin 3-p-hydroxy benzoyl sophoroside-5-glucoside, not only exhibited significant thermal stability but also had a role as a chemotherapeutic agent (Xu et al., 2015). In the present study, the contents of cyanidin 3-p-hydroxy benzoyl sophoroside-5-glucoside were significantly higher by 58.65% in zi8-D than zi8-X. These results provide basis of further research which can assist in the modulation of the proportion of anthocyanins by increasing the tinctorial intensity and stability of PFSP by improving the methods of cultivation.

The starch that isolated from the sweetpotato serves as an excellent raw material and food additive in the processing industry. Especially amylose has always been an important marker in determining starch quality (Sun et al., 2018). The starch from the sweetpotato cultivars exhibits a wide range of variations in terms of production and quality, and this is highly influenced by the environmental factors within a certain range (Yong et al., 2018). Previously, many researchers have observed that amylose content tends to increase with the rise in temperature (Norbert and Jutta, 1996; Tester et al., 1999; Tester and Karkalas, 2001; Yusuph et al., 2003; Kong et al., 2015). The amylose content in the present investigation revealed that zi8-X had 15.54% higher content than zi8-D (*p* < 0.05), which may be due to higher precipitation and higher temperature of Xuzhou. It can be inferred from the above data that amylose contents can be regulated by following the desirable cultivation procedure.

The physicochemical properties of starch were also analyzed both environmental conditions. The average DG of zi8-X and zi8-D was 16.88 and 19.56 μm, respectively, which showed that the DG was not only genotypic but also environment-dependent. By comparing the differences between the two regions, it might be deduced that low temperature and drought stress may contribute to an increase in DG.

Previously, a significant positive correlation was demonstrated between the peak viscosity and concentration of amylopectin (Kaur et al., 2016). In the present study, peak viscosity of zi8-D was significantly higher than zi8-X, and the results were consistent with amylopectin contents. Drought and low temperature might lead to the accumulation of amylopectin, which further affects the peak viscosity. Therefore, the viscosity of starch can be modulated through cultivation according to the production needs. The starch structure of “Xuzhishu8” was studied in this research and PDI and conformation plot results could provide a reference for the industrial application of PFSP starch.

## Data Availability Statement

The original contributions presented in the study are included in the article/Supplementary Material, further inquiries can be directed to the corresponding author/s.

## Author Contributions

HY: conceptualization, software, formal analysis, writing—original draft preparation, and visualization. XW: methodology. HY, MM, and MK: validation. HY and WS: investigation. WT, HY, and RG: resources. CL and HY: data curation. HY, MQA, and MHA: writing—review and editing. QL: supervision and funding acquisition. YZ and ZL: project administration. All authors have read and agreed to the published version of the manuscript.

## Conflict of Interest

The authors declare that the research was conducted in the absence of any commercial or financial relationships that could be construed as a potential conflict of interest.

## Publisher’s Note

All claims expressed in this article are solely those of the authors and do not necessarily represent those of their affiliated organizations, or those of the publisher, the editors and the reviewers. Any product that may be evaluated in this article, or claim that may be made by its manufacturer, is not guaranteed or endorsed by the publisher.
