# Supplementary material for: Comparative Analysis of Anthocyanin Compositions and Starch Physiochemical Properties of Purple-Fleshed Sweetpotato “Xuzishu8” in Desert Regions of China
Source: Front Plant Sci. 2022 Apr 12;13:841969. doi: 10.3389/fpls.2022.841969 (PMC9039657; doi:10.3389/fpls.2022.841969)
Supplement: Supplementary file 1 [file Data_Sheet_1.doc]

Supplementary Table 1 Soil composition in Xuzhou and Dalad Banner (mean ± SE)

|  | Soil salt ‰ | pH | Alkaline nitrogen(mg/kg) | Available phosphorus (mg/kg) | Available  potassium (mg/kg) | Organic matte (g/kg) |
| --- | --- | --- | --- | --- | --- | --- |
| Xuzhou | 0.53B±0.1 | 7.89A±0.1 | 70.65A±1.32 | 16.55A±3.45 | 94.25A±12.28 | 13.95A±0.91 |
| Dalad Banner | 0.31A±0.31 | 7.92A±0.11 | 35.22B±0.01 | 0.47B±1.44 | 49.89B±2.17 | 5.12B±1.28 |

Supplementary Table 2 Temperature and precipitation of Xuzhou and Dalad Banner in 2019 and 2020

| Year | Month | Xuzhou | | | Dalad Banner | | |
| --- | --- | --- | --- | --- | --- | --- | --- |
|  |  | monthly mean maximum temperature /℃ | monthly mean minimum temperature /℃ | Precipitation / mm | monthly mean maximum temperature /℃ | monthly mean minimum temperature /℃ | Precipitation / mm |
| 2019 | Jan. | 5 | -2 | 17.6 | 0 | -12 | 6.3 |
|  | Feb. | 7 | 0 | 20.5 | 4 | -9 | 0 |
|  | Mar. | 16 | 5 | 36 | 13 | -2 | 0 |
|  | Apr. | 21 | 10 | 47.1 | 22 | 7 | 17.3 |
|  | May | 27 | 16 | 65.5 | 23 | 9 | 9.8 |
|  | Jun. | 31 | 21 | 106.8 | 28 | 16 | 57.9 |
|  | Jul. | 33 | 22 | 241 | 29 | 18 | 82.3 |
|  | Aug. | 30 | 23 | 132.6 | 28 | 16 | 61.3 |
|  | Sep. | 28 | 19 | 72.3 | 25 | 12 | 41.4 |
|  | Oct. | 21 | 12 | 51.5 | 17 | 4 | 18.3 |
|  | Nov. | 15 | 6 | 26.7 | 8 | -1 | 7.1 |
|  | Dec. | 9 | 0 | 14 | 2 | -8 | 0 |
|  | Total | / | / | 831.6 | / | / | 301.7 |
| 2020 | Jan. | 6 | 0 | 55.2 | 2 | -9 | 0 |
|  | Feb. | 11 | 2 | 21.5 | 7 | -6 | 8.6 |
|  | Mar. | 16 | 6 | 36.5 | 14 | 0 | 9.5 |
|  | Apr. | 21 | 9 | 42.0 | 19 | 4 | 0 |
|  | May | 27 | 17 | 45.9 | 25 | 11 | 29.7 |
|  | Jun. | 29 | 21 | 102.6 | 30 | 16 | 88.5 |
|  | Jul. | 28 | 22 | 190.4 | 30 | 19 | 96.7 |
|  | Aug. | 32 | 25 | 150.8 | 28 | 17 | 58.5 |
|  | Sep. | 28 | 19 | 62.7 | 23 | 11 | 0 |
|  | Oct. | 20 | 11 | 27.6 | 15 | 3 | 0 |
|  | Nov. | 14 | 6 | 25.9 | 7 | -2 | 8.5 |
|  | Dec. | 6 | -2 | 0 | -1 | -10 | 7.6 |
|  | Total | / | / | 761.1 | / | / | 307.6 |

| **NO.** | **Component** Name | **RT** | **M**W | **NO.** | **Component** Name | **RT** | **M**W |
| --- | --- | --- | --- | --- | --- | --- | --- |
| 1 | Cyanidin 3-(6’’-caffeoyl sophoroside)-5-glucoside | 8.77 | 935 | 12 | Peonidin 3-p-hydroxybenzoyl sophoroside-5-glucoside | 7.16 | 907 |
| 2 | Cyanidin 3-p-hydroxybenzoyl sophoroside-5-glucoside | 5.95 | 893 | 13 | Peonidin 3-sophoroside-5-glucoside | 3.89 | 787 |
| 3 | Cyanidin3-(6’’-caffeoyl-6’’’-feruloylsophoroside)-5-glucoside | 10.53 | 1111 | 14 | Peonidin 3-feruloyl-p-caffeoylsophoroside-5-glucoside | 11.39 | 1125 |
| 4 | Cyanidin 3-sophoroside-5-glucoside | 2.97 | 773 | 15 | Peonidin 3-(6’’-feruloylsophoroside)-5-glucoside | 9 | 963 |
| 5 | Cyanidin 3-(6’’,6’’’-dicaffeoylsophoroside)-5-glucoside | 9.68 | 1097 | 16 | Peonidin 3-(6’’,6’’’-dicaffeoylsophoroside)-5-glucoside | 10.88 | 1111 |
| 6 | Cyanidin 3-(6’’-feruloylsophoroside)-5-glucoside | 7.83 | 949 | 17 | Peonidin 3-caffeoylsophoroside-5-glucoside | 9.59 | 949 |
| 7 | Cyanidin 3-(6’’-p-coumarylsophoroside)-5-glucoside | 9.37 | 919 | 18 | Pelargonidin 3-sophoroside-5-glucoside | 3.63 | 757 |
| 8 | Cyanidin 3-caffeoylsophoroside-5-glucoside | 8.75 | 935 | 19 | Cyanidin 3,5-diglucoside | 5.09 | 611.15955 |
| 9 | Cyanidin 3-caffeoyl-p-coumarylsophoroside-5-glucoside | 10.2 | 1081 | 20 | Peonidin 3-(6¢¢-*p*-coumarylsophoroside)-5-glucoside | 9.20 | 933.2637 |
| 10 | Peonidin 3-caffeoyl-p-hydroxybenzoyl-sophoroside-5-glucoside | 11.05 | 1069 | 21 | Peonidin 3-feruloyl-*p*-coumarylsophoroside-5-glucoside | 12.67 | 1109.3105 |
| 11 | Peonidin 3-caffeoylsophoroside-5-glucoside | 9.64 | 949 |  |  |  |  |

Supplementary Table 3 Component name, retention time (RT) and molecular weight (MW) of the 21 standards
